# The Influence of Hemp Fibers (*Cannabis sativa* L.) on the Mechanical Properties of Fiber–Gypsum Boards Reinforcing the Gypsum Matrix

**DOI:** 10.3390/polym16182644

**Published:** 2024-09-19

**Authors:** Adrian Trociński, Marek Wieruszewski, Monika Bartkowiak, Dorota Dziurka, Radosław Mirski

**Affiliations:** 1 Department of Mechanical Wood Technology, Faculty of Forest and Wood Technology, Poznan University of Life Sciences, Wojska Polskiego 38/42, 60-627 Poznan, Poland; marek.wieruszewski@up.poznan.pl (M.W.); dorota.dziurka@up.poznan.pl (D.D.); 2 Department of Wood Chemical Technology, Faculty of Forest and Wood Technology, Poznan University of Life Sciences, Wojska Polskiego 38/42, 60-627 Poznan, Poland; monika.bartkowiak@up.poznan.pl

**Keywords:** gypsum, fiber–gypsum boards, hemp (*Cannabis sativa* L.)

## Abstract

The modern construction industry is looking for new ecological materials (available, cheap, recyclable) that can successfully replace materials that are not environmentally friendly. Fibers of natural origin are materials that can improve the properties of gypsum composites. This is an important issue because synthetic fibers (hardly biodegradable—glass or polypropylene fibers) are commonly used to reinforce gypsum boards. Increasing the state of knowledge regarding the possibility of replacing synthetic fibers with natural fibers is another step towards creating more environmentally friendly building materials and determining their characteristics. This paper investigates the possibility of manufacturing fiber–gypsum composites based on natural gypsum (building gypsum) and hemp (*Cannabis sativa* L.) fibers grown in Poland. The effect of introducing hemp fibers of different lengths and with varying proportions of mass (mass of gypsum to mass of fibers) into the gypsum matrix was investigated. The experimental data obtained indicate that adding hemp fibers to the gypsum matrix increases the static bending strength of the composites manufactured. The highest mechanical strength, at 4.19 N/mm^2^, was observed in fiber–gypsum composites with 4% hemp fiber content at 50 mm in length. A similar trend of increased strength was observed in longitudinal tension. Again, the composite variant with 4% fiber content within the gypsum matrix had the highest mechanical strength. Manufacturing fibers–gypsum composites with more than 4% hemp fiber content negatively affected the composites’ strength. Mixing long (50 mm) hemp fibers with the gypsum matrix is technologically problematic, but tests have shown a positive effect on the mechanical properties of the refined composites. The article indicates the length and quantity limitations of hemp fibers on the basis of which fiber–gypsum composites were produced.

## 1. Introduction

The requirements for the modern construction industry no longer result only from construction standards or national regulations. Consumers are increasingly looking for and deciding to purchase (despite the higher price) ecological materials. One of the most promising plants is *Cannabis sativa* L., which has been used and processed by people for several thousands of years in various ways. The plant derives its popularity from several factors, such as its ease of cultivation, adaptability to different habitats and climatic conditions, wide distribution area, and ability to use other plant parts. Hemp is grown for the seed, inflorescence, or hemp straw [1]. Within Europe, an apparent increase in the volume of hemp cultivation of 75% was observed between 2015 and 2019. The most recent data indicate that nearly 35,000 ha of hemp seed are cultivated in the EU, producing 152,820 tons of hemp straw per year [2]. In line with the increasing trend in hemp cultivation, there has also been growing interest from industry and the scientific community worldwide in the possibilities of utilizing and processing hemp straw, from which shives (the woody part of the stalk) and fibers are obtained. Some scientific reports suggest using hemp fibers to produce furniture boards [3,4]. Hemp fiber-based materials that are both acoustic and thermal insulators have also been manufactured [5,6,7]. The versatility and high natural mechanical strength of hemp fibers [8] have led to attempts to combine them with various materials as fillers and structure-stabilizing agents of the resulting material, e.g., polypropylene [9,10,11], cement [12], or gypsum [13]. In particular, attempts to combine hemp fibers with different gypsums are an exciting research and technological topic. The vast possibilities of manipulating the technical parameters of the resulting composites result in a great interest in finished products in the construction industry. In addition, it should be noted that fiber–gypsum composites can be regarded as environmentally friendly materials. The following factors support this statement:

- Hemp absorbs large amounts of CO_2_ from the atmosphere during the growing season [11,14] and can absorb heavy metals from the soil in post-mining areas [15]. A significant advantage of the plant is its relatively low energy intensity during processing, making it possible to use it to produce so-called ‘greenhouses’.
- Natural gypsum, whose reserves in Poland have been determined to be 261 million MG [16], is extracted from mineral resources (calcium sulfate dihydrate) in the form of gypsum rock. Gypsum is a widely used material that is safe for the environment and can be recycled.

Depending on the place of origin, the initial origin and even the place of origin in the stem, hemp fibers may have different mechanical and even electrical properties [17]. Taking into account the growing trends in the cultivation of hemp in Poland [18] (mainly for the cosmetics and textile industries), the limited possibilities of using link fibers with gypsum obtained in the country are limited. Hemp fibers input to the matrix have a free role in the structure, just like innovative glass [19] or polypropylene [20] units, but as a natural material it is much more problematic.

In the scientific literature, long hemp fibers are usually considered to be 20 mm [21]. Introducing longer fibers into gypsum matrices (as in the article) is difficult, but possible. Thus, the influence of the length of hemp fibers on the mechanical properties of composites based on a given dimensional group was determined. An additional novelty in the article is the determination of the maximum share of hemp fibers that can be introduced into the plaster slurry. In the literature, the most common fiber content introduced into the gypsum slurry is 1–2% [22]. Such a small content of fibers introduced into the gypsum matrix results from the large volume difference between gypsum and fibers. Increasing the share of fibers in the matrix significantly affects the properties of the produced composites, as shown in the presented results.

This paper presents the characteristics of the fiber–gypsum composite. The relationship between the mass content of the filler hemp fibers (*Cannabis sativa* L.) and the matrix made from natural gypsum has been determined. The effect of the length of the hemp fibers used to manufacture the composites on the mechanical properties of the resulting materials was also investigated.

## 2. Materials and Methods

### 2.1. Materials

The matrix of the fiber–hemp composite was made from building gypsum (CaSO_4_ +1/2 H_2_O of the β variety). Building gypsum is obtained by partially dehydrating natural gypsum rock (calcium sulfate dihydrate) from an open-pit gypsum mine in southern Poland (Nida Valley, Leszcze, Poland). It is characterized by a fast curing time and good mechanical properties [23]. According to the producer’s specifications, the essential characteristics of the material are a water–gypum ratio (W-G) of 0.6 L–1 kg, it starting to set after about 3 min, this setting ending after 30 min, and its exhibiting an exothermic hardening reaction up to 50 °C.

**Figure 1:**
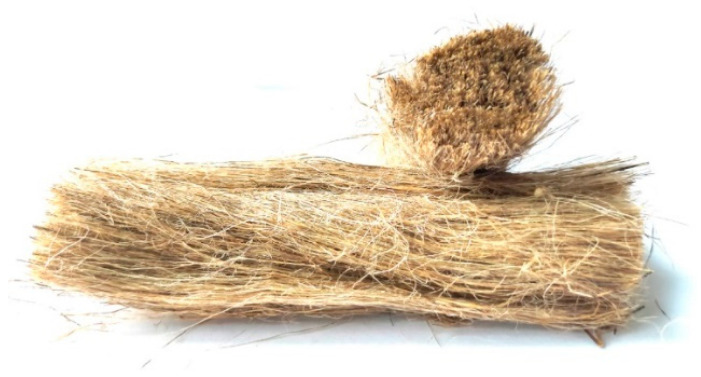
Hemp fibers used for testing—own photo.

The fibers used as filler were hemp seed fibers (*Cannabis sativa* L.) of the Białobrzeska variety grown in the Wielkopolska region. The fibers were purchased from the LenKon company in Stęszew (Experimental Department of the Institute of Natural Fibers and Herb Plants, Poznań, Poland). The producer cut the fibers obtained into the following lengths: 5 mm, 20 mm, 50 mm, and 100 mm, and then pre-pressed to vent the fiber mass (Figure 1).

### 2.2. Technological Process of Manufacturing Fibre–Gypsum Composites

The hemp fibers were dispersed and dried to a moisture content of approximately 2–3% before being introduced into the gypsum matrix. This action enables the hemp fibers to be introduced more uniformly into the gypsum slurry during the primary process. The manufacturing process of the fiber–gypsum composites started with preparing a gypsum slurry according to EN 196-1:2016-07 [24], with a water–gypsum ratio of 0.6 L–1 kg. Depending on the variant of the composite to be manufactured, hemp fibers of a certain length or mass were then introduced into the prepared gypsum slurry. The prepared gypsum–fiber slurry mixture was introduced into the matrix, forming a 255 mm × 355 mm board. The fiber–gypsum composites were cured in the matrix for 2 h and then subjected to drying for 12 h at 55 °C. The final step was to cut specimens with dimensions adapted to the normative requirements for the tests carried out.

### 2.3. Methods of Testing

#### 2.3.1. Measurement of the Setting Time (Beginning and End Method)

The test was carried out based on EN 13279-2 [25], using a tool in the form of a knife with the following dimensions: a length of 100 mm, a width of 16 mm, and the thickness of the upper blade at 1 mm, with a wedge-shaped cross-section, smooth glass plates (base), a spatula, a mixing container, and an electronic stopwatch. Each variant was tested in a min. of 3 trials. The beginning of the setting time, as defined by the standard, is considered to be the time expressed in minutes, after which the edges of the knife-cut ‘cake’ of gypsum slurry do not merge into their original state. The gypsum setting time was calculated according to the following formula:

T_i_ = t_1_ − t_0_ [min] (1)

where the following holds:

t_1_—the beginning of setting time; t_0_—test start time.

#### 2.3.2. The Determination of the Density of the Composites

The determination of density consisted of accurately measuring linear dimensions using an electronic caliper (Sylvas2tic IP67) and a thickness gauge based on PN-EN 323:1999 [26]. The samples were weighed with an accuracy of 0.01 g using an electronic weighing (Radwag). The density was calculated based on the following formula:

(2) $$\sigma =\frac{m}{b_{1}\times b_{2}\times t}\times 10^{6}[kg/m^{3}]$$

where the following holds:

m—sample weight; $b_{1}$—sample length; $b_{2}$—sample width; t—sample thickness.

#### 2.3.3. The Determination of the Radiological Density of the Manufactured Composites

A Hyperion tomograph with Irys 2.0 computer software was used for measurement. The tests were conducted in an 80 mm × 80 mm operating field and at the highest measurement quality. Random samples of 50 mm × 50 mm were taken for testing. In each selected sample, a minimum of 10 layers were measured on the surface, on which HU values were calculated. Each measurement was carried out on an area not less than 2000 mm^2^.

#### 2.3.4. Evaluation of Mechanical Properties

The determination of static bending strength and bending modulus was carried out by PN-EN 310:1999 [27]. The test specimens were cut using a format saw to the dimensions specified by the standard (250 mm × 50 mm × 8 mm). Each of the tested variants was tested in at least 10 tests. The longitudinal tensile strength determination was determined on specimens of 200 mm × 25 mm (b) × 8 mm (t) dimensions. The force used to break the specimen ($F_{max}$) was applied at a constant speed so that failure occurred within 60 ± 5 s of the start of stretching. The strength was calculated using the following formula:

(3) $$P_{t}=\frac{F_{max}}{t\times b}\;[N/mm^{2}]$$

where the following holds:

$F_{max}$—breaking load; t—the thickness of the sample; b—the width of the sample.

Depending on the variant, the specimens were subjected to a destructive force until the gypsum matrix broke (gypsum composites) or until the fibers broke and were pulled out of the matrix (fiber–gypsum composites). The test was conducted using a Tinius Olsen H10KT testing machine on specimens with a 2–3% moisture content.

#### 2.3.5. Statistical Analysis

The test results were statistically analyzed using Statistica 12.0 software (StatSoft Inc., Tulsa, OK, USA).

## 3. Results and Discussion

**Figure 2:**
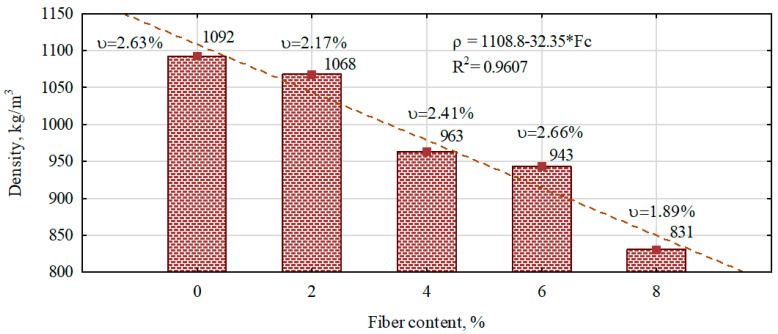
The influence of the proportion of hemp fibers on the density of manufactured composites.

The gypsum boards and fiber–gypsum composites were manufactured using a constant technological process, a constant water–gypum ratio, and a constant fiber length (50 mm) and subjected to a drying process immediately before the test. The difference between the variants concerned the change in hemp fiber content introduced into the gypsum matrix. The density of the gypsum boards and fiber–gypsum composites produced is shown in Figure 2. The introduction of a 2% hemp fiber content resulted in a 4.1% decrease in the density of the gypsum boards. Further increases in the proportion of hemp fibers introduced into the fiber–gypsum composites resulted in a density reduction of 11.8%, 13.6%, and 23.9%, respectively.

Also, as compared to other scientific works, regardless of the form (hemp powder and fibers 8 mm–12 mm [28,29,30] in height) in which hemp fibers are introduced into the gypsum matrix, the density of the refined composite decreases.

**Figure 3:**
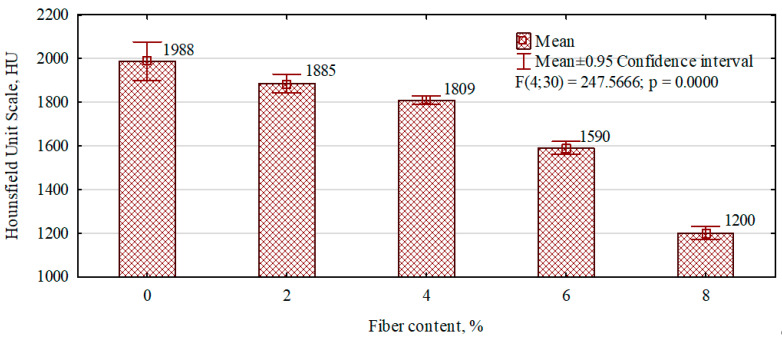
The influence of the content of hemp fibers on the radiographic density of the composites manufactured.

The radiographic density of fiber–gypsum composites, produced based on building gypsum, decreases with the amount of hemp fibers introduced into their structure (Figure 3). ANOVA analysis shows that the changes are statistically significant (F(4, 30) = 131.21, *p* = 0.0000).

**Figure 4:**
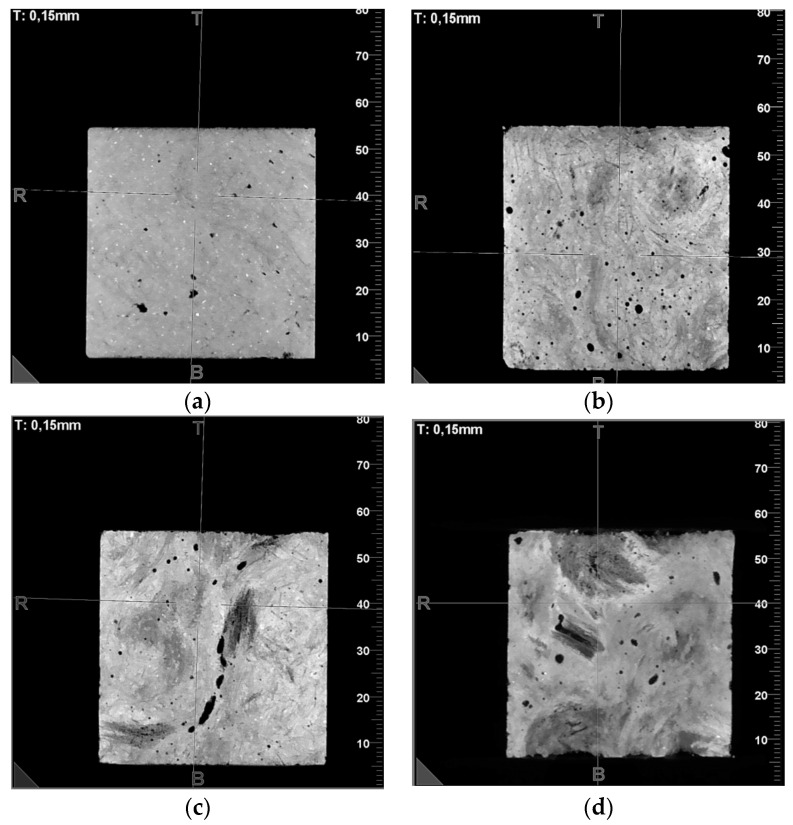
Photographs of an internal cross-section of fiber–gypsum composites manufactured based on building gypsum with the following hemp fiber contents: (**a**)—2%; (**b**)—4%; (**c**)—6%; (**d**)—8%.

The highest radiographic density was found in gypsum boards, where no fibers were introduced into the internal structure. Introducing 2 per cent hemp fibers resulted in only a 5 per cent reduction in the HU radiographic density relative to the gypsum board. More significant differences were observed for composites in which fibers were introduced in larger quantities. Thus, for quantities of 4%, 6%, and 8%, there was a decrease in density relative to gypsum boards of 9.5%, 21%, and 39.7%, respectively. The significant reduction in radiant density for the variants with 6% and 8% hemp fiber content in the structure may be due to an increase in the number and size of voids, i.e., air enclosed in the cured gypsum matrix. This phenomenon was observed in the photographs of the inner cross-sectional layers of the individual variants (Figure 4).

**Figure 5:**
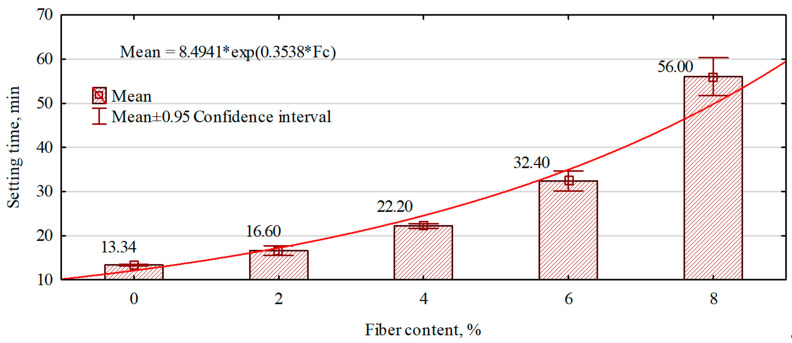
The influence of the amount of hemp fibers introduced on the setting time of the gypsum slurry.

The setting time of gypsum slurry is one of the most critical parameters for manufacturing gypsum boards and fiber–gypsum composites. This parameter can affect, among other things, the mechanical properties of the materials produced or generate defects in the hardened board structure, such as cracks. The setting time of joints in the case of gypsum boards and fiber–gypsum composites is shown in Figure 5. The dependence of the increase in the setting time of the gypsum matrix with an increase in the amount of hemp fibers introduced into the gypsum slurry can be seen. Hemp fibers are a hydroscopic material that can absorb and release water. When combined with the gypsum slurry during the composite manufacturing process, the fibers absorb water from the slurry. The saturated fibers take longer to give up water from the surrounding gypsum matrix, which will likely extend the setting process. This assumption would confirm the significantly increased hardening time of the composite, with growing amounts of fibers introduced.

**Figure 6:**
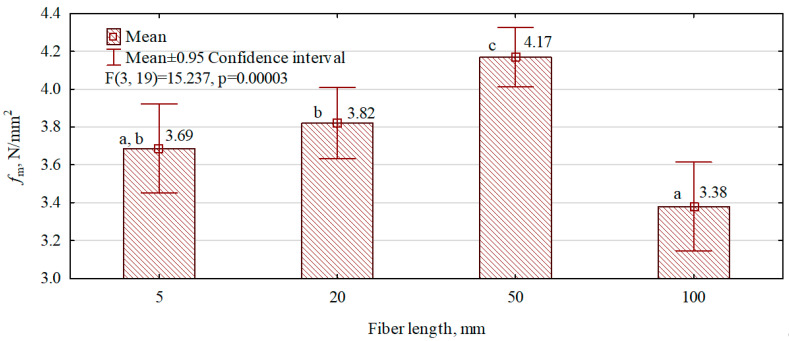
The influence of fiber length on the static bending strength of manufactured fiber–gypsum composites. (a–c letters indicate homogeneous groups).

The variants of fiber–gypsum composites to be tested were manufactured using a constant technological process and identical material ratio (fiber content 2%, W/G ratio = 0.6/1). The only variable factor was the length of hemp fibers introduced into the internal structure. The study aimed to identify the optimum size of the fibers, which are assumed to work as ‘reinforcement’ in the gypsum matrix. The results are presented in Figure 6. A two-factor ANOVA analysis was performed to verify this effect. The highest static flexural strengths were exhibited by composites in which 50 mm long fibers were introduced into the structure. This variant was the only one to achieve strengths above 4 N/mm^2^. Composites in which fibers were 5 mm and 20 mm in length which were introduced into the structure only slightly increased the static flexural strength of the composites compared to gypsum boards. The composites with the longest introduced hemp fibers (100 mm) had the lowest strength. The significant decrease in strength is due to the technological difficulties encountered in introducing such long fibers and distributing them evenly in the gypsum slurry. The hemp fibers can concentrate and tighten around the mixer, which increases the processing time and reduces the uniformity of distribution in the matrix.

**Figure 7:**
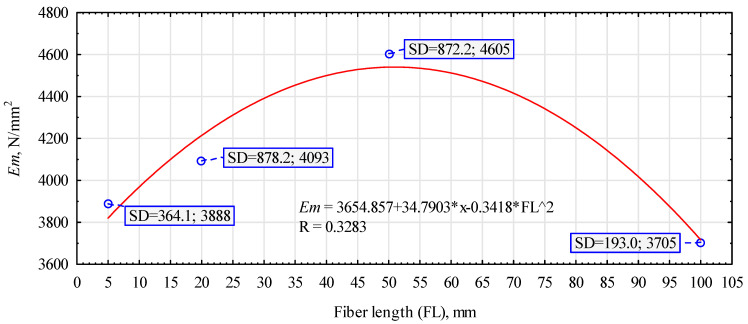
The influence of the length of hemp fibers on the modulus of elasticity of the composites manufactured.

A similar trend was observed for the modulus of elasticity (Figure 7). Again, the highest modulus of elasticity is characterized by the composite in which hemp fibers with a length of 50 mm were introduced into the structure. Variants with shorter fibers have an average modulus of elasticity 12% lower. On the other hand, the introduction of long fibers (100 mm) reduces the value of the modulus by almost 20%.

**Figure 8:**
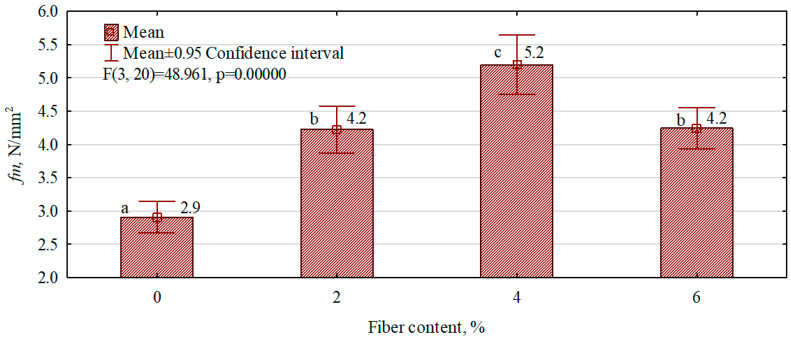
The influence of the content of hemp fibers introduced into the gypsum matrix on the static bending strength of the manufactured composites. (a–c letters indicate homogeneous groups).

On the other hand, the results of testing the effect of the amount of hemp fibers introduced on the bending strength of the manufactured gypsum and fiber–gypsum boards are presented in Figure 8. The boards were manufactured while maintaining constant parameters of the technological process and a constant material ratio (W/G ratio = 0.6/1). The variable factor was the hemp fiber dry weight to gypsum dry weight ratio. Fibers of approximately 50 mm were introduced into all types of composites manufactured. The boards with 4% hemp fibers introduced into the structure had the highest strength. In this case, an increase of up to 75.5% in the property was observed for the gypsum board (reference). Composites with 6% fiber content have an almost identical strength to those with 2%. The strength of both board types is more than 40% higher than that of the reference (gypsum) board.

It should be noted, however, that boards with 6% fiber content have a lower density. The introduction of 8% fiber, however, reduces the bending strength of the composite below that of the gypsum boards. The likely reason for the significant decrease in strength is the insufficient gypsum matrix relative to the amount of fibers introduced. The results of other scientists also indicate an increase in the bending strength of gypsum boards after introducing hemp fibers into the gypsum matrix in various forms [31,32,33].

**Figure 9:**
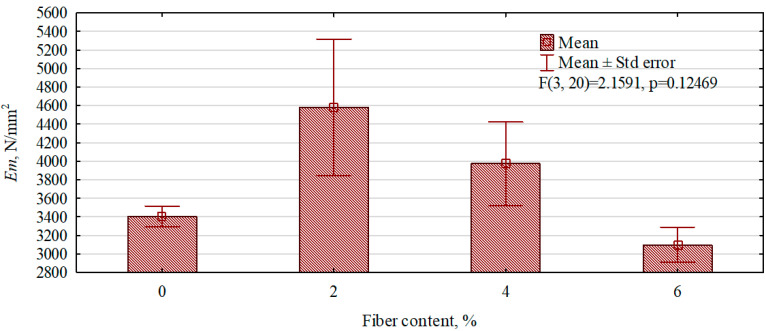
The influence of the content of hemp fibers introduced into the gypsum matrix on the modulus of elasticity of the composites produced.

Figure 9 shows the effect of the amount of hemp fibers introduced into the gypsum matrix on the modulus of elasticity of the manufactured gypsum and fiber–gypsum boards. Fiber–gypsum composites with a fiber content of 2% showed the highest value for the modulus of elasticity, showing a 25.4% increase compared to gypsum boards, and increasing the fiber content to 4% results in an increase of only 8.5%, despite showing the highest static bending strength of these boards. In fiber–gypsum composites with a fiber content of more than 4%, a decrease in the modulus of elasticity below that of boards without fibers in the structure was observed.

**Figure 10:**
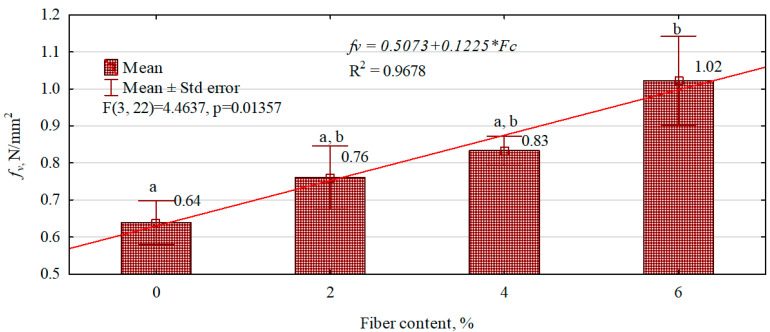
The effect of hemp fiber content introduced into the gypsum matrix on the tensile strength parallel to the planes of the manufactured composites. (a–b letters indicate homogeneous groups).

Studies conducted on the effect of the amount of hemp fibers introduced into the gypsum matrix on the tensile strength parallel to the planes show that with an increase in their amount, the strength of the composites increases linearly (Figure 10), from 20% to as much as over 60%. The most significant increase in tensile strength is seen in composites in which 6% of the fibers have been introduced, even though, in this case, the fibers are least evenly distributed in the composite structure.

**Figure 11:**
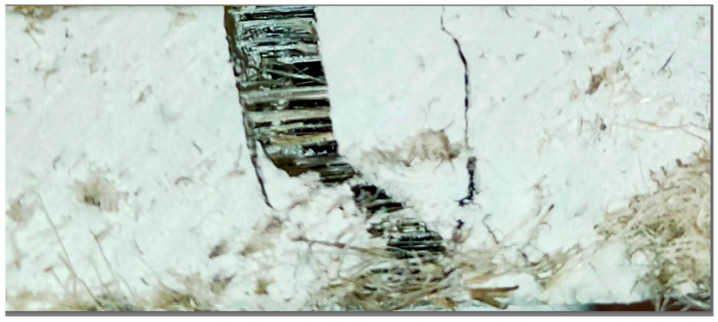
An example of damage after the tensile test.

Although the uniformity of the hemp fiber distribution appears to be a key factor influencing the mechanical strength of fiber–gypsum composites, the amount of fibers oriented parallel to the acting force may be more critical for tensile strength (Figure 11).

**Figure 12:**
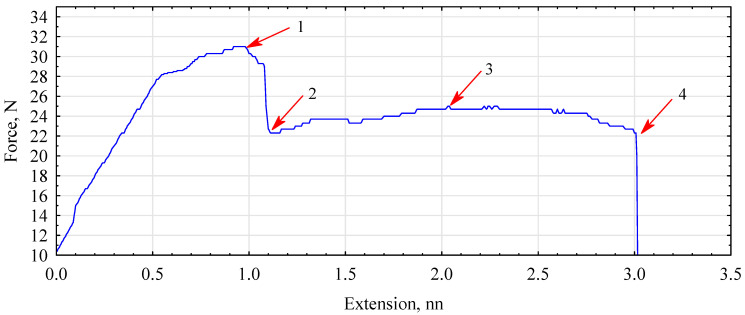
The graph of the static bending curve tested on a fiber–gypsum composite containing 4% hemp fibers.(1—max. force, 2—destruction of the plaster structure, 3—the fibers take over the load, 4—complete destruction).

The process of measuring the static bending strength of fiber–gypsum composites is presented in Figure 12. The testing of individual variants can be divided into four stages: (1) The stage in which the maximum destructive force is applied. However, here the plaster matrix cracked, but the tested variant did not completely break down. (2) The area where hemp fibers embedded in the matrix stop the further destruction process. (3) As the force increases, the graph shows vibrations resulting from the tearing of larger clusters of hemp fibers from the gypsum matrix. (4) The stage of complete sample disruption.

In the case of mechanical tests, the structure of the gypsum–hemp composite sample was damaged in the form of cracks.

The first observed crack concerns the disruption of the gypsum matrix structure. The further application of force causes the hemp fibers embedded in the matrix to be torn out perpendicular to the place of the crack in the plaster matrix.

## 4. Conclusions

The research was limited to the level of verification of the properties of fiber–gypsum composites present due to the combination of natural hemp fibers and the gypsum matrix. The influence of the length of hemp fibers and the influence of the fiber content introduced into the gypsum matrix on the physical and mechanical properties of the produced composites was determined. The research was conducted under laboratory conditions, which can alter the level of fiber distribution in the composite matrix.

The density of fiber–gypsum composites, including radiographic density, decreases with increasing hemp fiber content. A decrease in the density of gypsum composites, as reported in the literature, is observed when both natural [28] and synthetic fibers are introduced into the matrix [29,30].

The setting time of gypsum joints depends on the size of the water–gypum coefficient and the content of hemp fibers introduced. The hemp fibers take longer to give up moisture than the gypsum matrix, increasing the manufactured composites’ setting time. The above relationship aligns with the observations of BaBu and Ratnam [28]. Indeed, these authors noted the relationship between the content of hemp fibers introduced into the gypsum matrix and the ability of the fibers to absorb water from the densifier, which is thought to affect the extension of the curing time of the composites manufactured.

Hemp fibers over 100 mm were unsuitable for introduction into the gypsum matrix. Favorable lay-ups were obtained using fibers 20 mm–50 mm in length. However, the amount of fiber introduced should be at most 4%. Further increases in the fiber content of the composites manufactured resulted in a decrease in static bending strength.

The length of the hemp fibers on which the fiber–gypsum composites were manufactured has a negligible effect on the static bending strength. The highest static bending strengths were those of the boards produced with fiber lengths of 50 mm.

The tensile strength parallel to the planes of the fiber–gypsum composites increases with increasing hemp fiber content in the board structure. This phenomenon can be attributed to a more uniform distribution of hemp fibers within the gypsum matrix. This finding can be confirmed by observing the tested samples’ failure process. Despite the crack of the gypsum matrix, hemp fibers in fiber–gypsum composites can still carry the load until they are entirely torn out of the matrix. The manufactured fiber–gypsum composites result in a material with good mechanical properties, an interesting “raw” appearance, and are easy to mechanically process. Despite the lack of resistance to water and moisture, fiber–gypsum composites can be used in dry construction and constitute a good material for creating interior partitions.

## Data Availability

The original contributions presented in the study are included in the article, further inquiries can be directed to the corresponding author.
